# Screening for next generation vaccines in human immune organoids

**DOI:** 10.1093/immhor/vlaf075

**Published:** 2025-12-30

**Authors:** Guangbo (Bill) Chen, Ashley Smith, Joel Lee, Priscilla Kang, Oviya Siva, Mark M Davis

**Affiliations:** Department of Obstetrics and Gynecology, Medical College of Wisconsin, Milwaukee, WI, United States; Center for Immunology, Medical College of Wisconsin, Milwaukee, WI, United States; Department of Microbiology and Immunology, Medical College of Wisconsin, Milwaukee, WI, United States; Hematopoiesis and Immunobiology Program, Versiti Blood Research Institute, Milwaukee, WI, United States; Department of Obstetrics and Gynecology, Medical College of Wisconsin, Milwaukee, WI, United States; Center for Immunology, Medical College of Wisconsin, Milwaukee, WI, United States; Department of Obstetrics and Gynecology, Medical College of Wisconsin, Milwaukee, WI, United States; Center for Immunology, Medical College of Wisconsin, Milwaukee, WI, United States; Department of Obstetrics and Gynecology, Medical College of Wisconsin, Milwaukee, WI, United States; Center for Immunology, Medical College of Wisconsin, Milwaukee, WI, United States; Department of Obstetrics and Gynecology, Medical College of Wisconsin, Milwaukee, WI, United States; Center for Immunology, Medical College of Wisconsin, Milwaukee, WI, United States; Institute for Immunology, Transplantation and Infection (ITI), Stanford University School of Medicine, Palo Alto, CA, United States; Department of Microbiology and Immunology, Stanford University School of Medicine, Palo Alto, CA, United States

**Keywords:** immune models, high-throughput screening, influenza viruses, cytokine adjuvants

## Abstract

The development of effective vaccines against emerging infectious diseases, including avian influenza strains such as H5N1 and H7N9, is hindered by the limited translational fidelity of animal models and the low throughput of traditional preclinical platforms. Human immune organoids are ex vivo, multicellular, lymphoid cultures derived from tonsil, or spleen tissue, offering a physiologically relevant and scalable system to model human germinal center biology and vaccine responses. We describe how tonsil and spleen organoids can support the rationale design of antigen and adjuvant for influenza vaccines. Coupled antigen strategies, which leverage pre-existing memory T cells, can significantly enhance responses to weak antigens, such as avian HA. Moreover, a cytokine screen performed in human immune organoids revealed distinct adjuvanticity profiles and mapped functional axes involving type I interferons and IL-12/IL-21 signaling. We propose a new paradigm: functional systems immunology, combining mechanistic perturbation in human immune organoids with high-dimensional immune profiling. This platform will enable the causal dissection of human immune regulation at a large scale. High-throughput screen of candidates will enable efficient vaccine designs.

## Introduction

The 1918 Spanish flu pandemic, caused by an H1N1 influenza virus, resulted in an estimated 50 million deaths worldwide and remains one of the deadliest infectious disease events in recorded history. Today, avian influenza viruses such as H5N1 and H7N9^1^ continue to pose serious pandemic threats due to their ability to infect humans, high mortality rates, and potential for genetic reassortment that could facilitate sustained human-to-human transmission.

**Figure:**
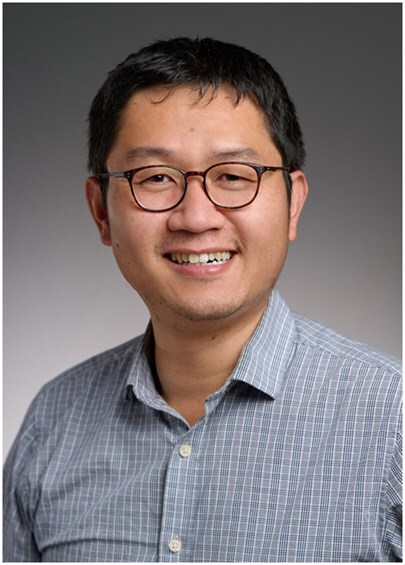
Dr. Guangbo (Bill) Chen is an Assistant Professor at the Medical College of Wisconsin. He worked as a Lilly Fellow of the Life Science Research Foundation in Mark Davis’s lab at Stanford. Dr. Chen pivoted to human immunology, adapting the novel spleen organoid system for high-throughput screening. This technology enables parallel testing of hundreds of organoids from a single donor, exemplified by his cytokine-adjuvant screen across multiple donors. Now, Dr. Chen is scaling the platform to systematically dissect human immune mechanisms and advance next-generation vaccines and immunotherapies.

By combining high-throughput functional assays with physiologically relevant human immune models, we can overcome key translational barriers that have hindered the development of vaccines. We argue that immune organoids provide an essential tool for rational vaccine design, enabling functional systems immunology studies that directly interrogate human immune mechanisms. Key terms and concepts used throughout this review are summarized in Box 1.

Box 1. Key concepts in influenza vaccinology **Organoid—**Ex vivo 3-dimensional culture derived from human lymphoid tissue that preserves key immune cell types and interactions, enabling functional modeling of vaccine responses.

**Antigenic drift—**Gradual accumulation of mutations in haemagglutinin (HA) and neuraminidase (NA) that allow circulating strains to evade pre-existing antibodies, necessitating regular vaccine updates.

**Antigenic shift—**Abrupt reassortment of gene segments in *influenza A*, producing novel HA and NA combinations that can lead to pandemics.

**Clade and cross-clade immunity—**Within a subtype, viruses are grouped into genetic clades based on HA sequence similarity. Cross-clade responses protect across multiple clades of the same subtype.

**Cross-subtype (heterosubtypic) immunity—**Protection that extends across different subtypes (such as H1 to H5), often mediated by antibodies recognizing conserved HA stem epitopes.

**Broadly neutralizing antibodies (bnAbs)—**Antibodies that neutralize diverse influenza strains by targeting conserved regions of HA, including the stem or receptor-binding site.

**Germinal center (GC)—**Specialized structure in secondary lymphoid tissue where B cells proliferate, mutate, and are selected to produce high-affinity antibodies with help from T follicular helper and follicular dendritic cells.

**Somatic hypermutation—**Process in which point mutations are introduced into immunoglobulin variable regions, generating antibody variants with altered affinity for antigen.

**Affinity maturation—**Selection of B cell clones producing higher-affinity antibodies that differentiate into long-lived plasma cells and memory B cells.

**Adjuvant—**A vaccine component that enhances immune activation by stimulating innate immune pathways.

**IIV and LAIV—**The inactivated influenza vaccine (IIV), administered intramuscularly, primarily elicits serum antibodies, whereas the live attenuated influenza vaccine (LAIV), delivered intranasally, induces interferon-rich mucosal and cellular responses.

Influenza viruses are divided into 4 types (A, B, C, and D) based on their internal protein characteristics. Influenza A viruses are further classified into subtypes based on the antigenic properties of their surface glycoproteins: hemagglutinin (HA) and neuraminidase (NA), resulting in the HxNx naming convention.^2^ In brief, 18 HA subtypes and 11 NA subtypes have been identified. Influenza A viruses are further grouped phylogenetically into group 1 and group 2 based on HA sequence similarities, while influenza B viruses are categorized into two antigenically distinct lineages, B/Victoria and B/Yamagata. Avian influenza viruses, such as H5N1 and H7N9, have historically caused sporadic but severe human infections with high mortality rates, underscoring the urgent need for effective pre-pandemic vaccines.^3^ While no avian flu vaccine is currently licensed for routine public use, several candidates have been developed using viral vectors, recombinant proteins, or virus-like particles that have demonstrated cross-clade protection.^4^ More generally, seasonal influenza vaccines are a cornerstone of global public health, having saved countless lives since their introduction in the 1940s by reducing the burden of annual epidemics.

A key limitation of current seasonal influenza vaccines is their variable efficacy, which can range from as low as 10% to a maximum of 60%.^5^ This inconsistency is partly due to antigenic drift and the time-consuming process of strain selection and vaccine production, which, in most cases, relies on growing viral stocks in chicken eggs.^6^ Another major limitation is strain preference, meaning that most people only make a significant response to one of the major flu types (H1, H3, or B). So even they are vaccinated with 3-4 strains in a typical seasonal formulation, they will most respond to the favored type. While for many decades this was thought to be the results of the first flu strain they were infected with as children, an idea known as “Original Antigenic Sin.”^7^ Original antigenic sin (OAS) describes the immune system’s tendency to preferentially recall antibody responses to the first influenza strain encountered, thereby strongly biasing subsequent responses toward that initial subtype rather than newly encountered variants. However, recent work by Mallajoysula et al. (2024) has demonstrated that host HLA genetics are a stronger driver of subtype bias to influenza viral strains. Moreover, it has been found that formulating a vaccine such that it provides abundant T cell help to the least favored strains can equalize the responses (see below). Furthermore, most licensed vaccines primarily induce strain-specific antibodies targeting the HA head, offering limited protection against drifted or pandemic strains. In the face of a pandemic threat, a next-generation influenza vaccine with much better efficacy is urgently needed.

High-throughput screening (HTS) has become a cornerstone of modern drug discovery by enabling the rapid evaluation of numerous compounds.^8^ Despite early skepticism, HTS has demonstrated substantial value, with success rates exceeding 60% and contributing to early drug discovery.^9^ Its scalability, versatility across target classes, and compatibility with both biochemical and phenotypic assays make it uniquely suited for drug development. However, high-throughput screening for vaccine development has been difficult due to lack of suitable model system.

While animal models mimic some aspects of the human immune response, there are also important differences. Moreover, the throughput of animal testing systems is considerably lower than that of cellular testing platforms. In contrast, in vitro immune function test systems often lack key immune cell subsets critical for orchestrating adaptive immune responses. Peripheral blood-derived systems, for instance, are deficient in lymphoid-resident cells, such as germinal center (GC) B cells, and T follicular helper (TFH) cells, which are essential components for the organization and function of the GC (Table 1).

**Table 1.** Comparison of different human or humanized immune models.

Feature	HuNSG mice^a^	Lymph node slice	PBMC organ-chip	Human immune organoid
Immune cell source	CD34^+^ stem cell	Lymph node	Human PBMC	Human spleen or tonsil
Human memory repertoire	**–**	**+**	**+**	**+**
Induced TFH/GC Formation	**–**	**–**	**– (T/B cluster)**	**+**
Somatic hypermutation	**–**	**–**	**– (AID expression)**	**+**
Affinity maturation	**–**	**–**	**–**	**+**
Long survival	**+**	**–**	**+**	**+**
Whole body test^b^	**+**	**–**	**–**	**–**
High-throughput screen	**–**	**–**	**–**	**+**
Examples of studies	^25–28^ ^,^ ^50^	^38^ ^,^ ^39^	^51^ ^,^ ^52^	^49^ ^,^ ^53^ ^,^ ^54^

a Recent THX mice demonstrated a much-improved GC response upon estrogen exposure.

b Lung or other organ damage test, animal/subject survival tests upon challenge.

– = Feature absent or not applicable; **+** = Feature present or supported.

The recent development of multicellular human lymphoid organ cultures (immune organoids), which transcend the limitations of both animal models and peripheral blood mononuclear cell (PBMC) cultures, represents a novel opportunity for high-throughput screening in vaccine research (Table 1).

## Current progress in designing next-generation influenza vaccines

There are several vaccine candidates specifically targeting the avian influenza strains. Examples include vaccines on DNA,^10^ vaccinia virus vector,^11^ or viruslike particle platforms.^12^ On the other hand, recent advances in immunology and structural biology have driven significant progress to enhance the breadth of protection.^4^ A major focus has been on targeting conserved regions of the HA protein, particularly the stem domain, which elicits broadly neutralizing antibodies capable of cross-subtype recognition. Novel vaccine constructs, such as headless HA immunogens and ferritin-based nanoparticles displaying stabilized HA stem regions, have demonstrated protective efficacy in animal models and are currently under clinical evaluation.^13^^,^^14^ Additionally, computationally optimized broadly reactive antigens (COBRA) and mosaic HA designs have demonstrated the ability to elicit cross-reactive responses against drifted strains.^15–17^ Beyond HA, other viral antigen, such as the matrix protein 2 ectodomain (M2e) has been explored as a universal vaccine antigen.^18–20^

## Mice as a low-throughput testing platform for antibody response

The generation of high-affinity, class-switched antibodies is a complex, multicellular process that unfolds within GCs, specialized microenvironments in secondary lymphoid tissues. Within GCs, B cells undergo somatic hypermutation and selection, guided by interactions with TFH cells, FDCs, and other cells.^21^ Evaluations of the multi-cellular process of antibody induction by vaccine candidates primarily rely on whole animal models (Table 1). However, immune cell function and regulation of immunoglobulin G (IgG) production differ significantly between mice and humans. For example, “TGN1412,” a CD28 superagonist antibody, was found to be safe in mice and a non-human primate model (*M. fascicularis*). Still, in humans, it caused a cytokine storm, demonstrating dramatic interspecies divergence in CD28 signaling thresholds and downstream cytokine cascades.^22^ Despite each species expressing 4 IgG subclasses, the relationships between these subclasses are not homologous. The cytokine signals governing class-switch recombination also diverge; for instance, IL-4 drives IgG1 switching in mice but favors IgG4 in humans.^23^

The immune cell repertoire differs between mice and humans, profoundly impacting antibody responses. Comparative analysis of human and mouse antibody responses to influenza H1 hemagglutinin reveals species-specific differences in epitope targeting.^24^ HA protein of H1 subtype has 5 major antigenic sites: Sa, Sb, Ca1, Ca2, and Cb.^24^ Research has shown that BALB/c mice infected with H1N1 viruses mount antibody responses dominated by the Sb and Ca2 sites on the hemagglutinin. In contrast, human antibody responses focus predominantly on antigenic sites surrounding the receptor binding domain (RBD), including Sb and Sa, with less consistent recognition of Ca2.

**Figure 1.**
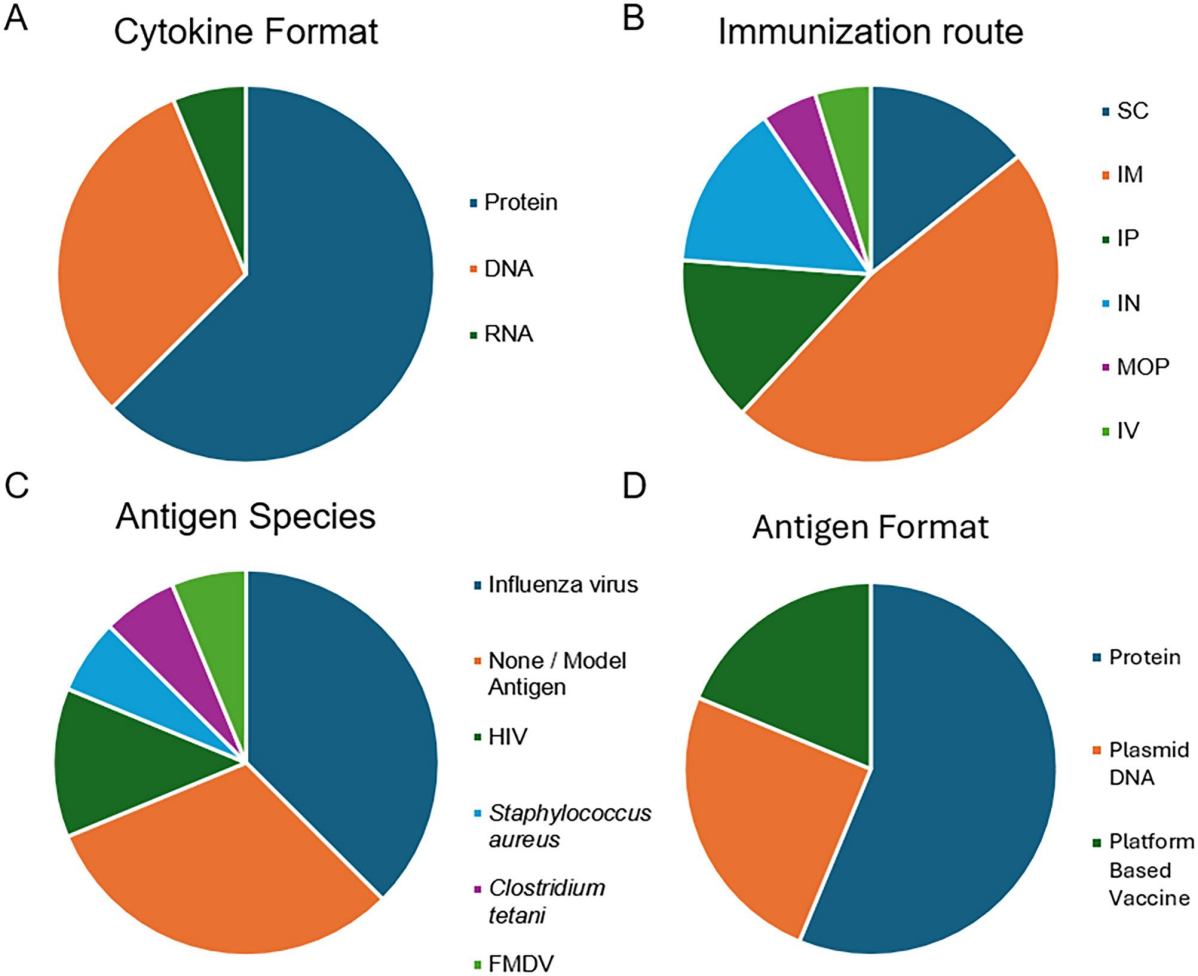
Heterogeneity of experimental conditions among studies demonstrating increased antibody responses with cytokine adjuvants. **(**A–D) Distribution of experimental parameters among mouse studies that reported increased antigen-specific antibody responses after cytokine addition. (A) Cytokine delivery formats: recombinant proteins, DNA plasmids, mRNA constructs, or cell-based expression systems. (B) Immunization routes: intramuscular (IM), subcutaneous (SC), intraperitoneal (IP), intravenous (IV), and intranasal (IN). (C) Antigen species: viral, bacterial, or model antigens used with cytokine adjuvants. (D) Antigen formats: most studies used protein-based antigens (including purified and fusion proteins), with others employing plasmid DNA or platform-based vaccines such as viral vectors, inactivated or split-virus preparations, and liposomal formulations. Because reported readouts (OD_450_, ng ml^−1^, fold-change, etc) varied widely, outcomes are summarized qualitatively by direction of effect. These distributions illustrate that reported antibody enhancements arise under diverse, non-standardized conditions, underscoring the need for harmonized experimental systems such as human immune organoids to evaluate cytokine adjuvants systematically.

The disparities between mice and humans underscore the importance of contextualizing murine findings when evaluating antibody production and T cell regulation in human immunity. HuNSG mice, constructed by grafting human hematopoietic stem cells into NSG mice, have contributed substantially to human immune modeling but face critical limitations in evaluating human antibody responses.^25^^,^^26^ HuNSG mice exhibit poor immune responsiveness, with limited B cell maturation, class-switch recombination, or somatic hypermutation, resulting in low levels of circulating human immunoglobulins and impaired antibody responses. Efforts to enhance these responses through cytokine knock-in approaches often result in abnormal, supraphysiological cytokine levels rather than physiologic immune function.^27^ Recently, a THX mouse exhibited robust human immune reconstitution, capable of mounting mature antibody responses, including class-switch recombination, somatic hypermutation, and clonal expansion of B cells, as demonstrated by the production of high-affinity IgG and IgA following immunization with NP-CGG, flagellin, or the coronavirus disease 2019 (COVID-19) mRNA vaccine.^28^ However, the need for exogenous estrogen conditioning to achieve this functionality may introduce confounding hormonal effects. Moreover, most mouse studies characterize the impact of only one or a few cytokines. These studies often employed varied delivery formats (DNA, RNA, protein, or cell-based), antigen types, and immunization routes (Fig. 1). We compiled published mouse cytokine co-adjuvant studies that reported increased antibody responses and categorized them by context to illustrate the diversity of conditions producing positive outcomes (Fig. 1). Because experimental readouts were highly heterogeneous, the figure summarizes qualitative trends rather than quantitative magnitudes. For example, in different models, IL-21 exerted variable effects ranging from increased IgG to no impact on IgG production.^29–34^ This variability highlights the challenge of generalizing findings across mouse studies and underscores the need for standardized, high-throughput human systems, such as the immune organoid platform.

## Modeling human GC reaction

Germinal centers (GCs) are specialized structures in secondary lymphoid tissues that arise after B cell activation by antigen.^21^ In the dark zone, B cells proliferate and undergo somatic hypermutation (SHM), introducing mutations into immunoglobulin genes that alter antigen-binding affinity. Mutated B cells then move to the light zone, where their receptors are tested for binding strength through interactions with FDCs and TFH cells. High-affinity clones are selected to either re-enter SHM cycles or differentiate into plasma cells and memory B cells, illustrating affinity maturation and the establishment of durable humoral immunity.

Various strategies have been explored to culture human lymphoid tissue-like environments in vitro. Compared with mouse models, these human cultures retain many human features, including a human T/B cell memory repertoire, which can profoundly impact the antibody responses. A primary goal of these works is to model the GC reaction in humans. A recent review provided a detailed comparison between these systems.^35^ Here, we will provide a brief summary of commonly used human systems. Co-cultures of CD4^+^ T cells and B cells demonstrated the role of TFH cells in supporting B cell maturation, particularly in the context of viral and autoimmune diseases.^36^^,^^37^ Tonsil or adenoid explants cultured on collagen sponges enabled analysis of HIV replication and early CD4^+^ T cell antigen recall responses, although their short lifespan limited the study of antibody production.^38^,39Additionally, 3D cultures using PBMCs embedded in ECM-based hydrogels or synthetic scaffolds allowed B cell differentiation into antibody-secreting cells, especially when supplemented with CD40L-expressing fibroblasts and cytokines.^40–42^ Microfluidic devices enhance cellular motility, antigen presentation, and T/B cell interactions under continuous media perfusion.^43–46^ These systems enabled the formation of lymphoid-like follicles and vaccine-specific antibody responses in vitro. However, PBMC-derived systems remain limited by the scarcity of lymphoid-resident cells such as FDCs or TFH cells. To compensate, strong immune stimulation (eg staphylococcal enterotoxin B^47^) and/or specialized conditions (such as an organ chip) are used to launch an antibody response.^48^ Despite these efforts, SHM, a hallmark of GC activity, is generally not observed in these models.^35^ Moreover, the complexity of setup renders PBMC-derived organoids less suitable for high-throughput applications.

Human tonsil organoids provide a physiologically relevant ex vivo platform that models both GC architecture and human vaccine responses.^49^ Upon stimulation with live attenuated influenza vaccine (LAIV), these organoids develop GC-like structures. Single-cell RNA-seq confirms transcriptional signatures of GC B cells, including genes involved in antibody secretion and BCR signaling.^49^ Functionally, LAIV-stimulated organoids show increased plasmablast frequencies (up to 30% of B cells) and produce influenza-specific IgG in most donors. ELISpot assays confirm the presence of antigen-specific antibody-secreting cells, and BCR sequencing reveals class-switched, clonally expanded HA-specific lineages with high mutation loads. When naive B cells are used as the sole source of B cells, organoids still produce high-affinity antibodies, demonstrating de novo affinity maturation.^49^ Importantly, the single-cell suspension format is adaptable for high-throughput screening (see below).

## Studying influenza vaccine response in human tonsil organoids

The ability to sustain long-term ex vivo culture and support GC-like features enables the use of tonsil organoids to study influenza vaccine responses.^49^ Different influenza antigen formats elicit distinct immune responses in terms of B and T cell activation, antibody production, and repertoire diversity.^53^ LAIV induces the highest frequencies of HA^+^ B cells and plasmablasts, as well as stronger neutralizing antibody responses, compared to inactivated influenza vaccine (IIV) or wild-type H1N1 virus. LAIV also drives broader antibody reactivity, including responses against influenza B and neuraminidase antigens, and promotes greater BCR diversity. In contrast, IIV responses are dependent on pre-existing memory B cells, show early class switching, and require memory CD4^+^ T cell help to generate HA-specific antibodies.

**Figure 2.**
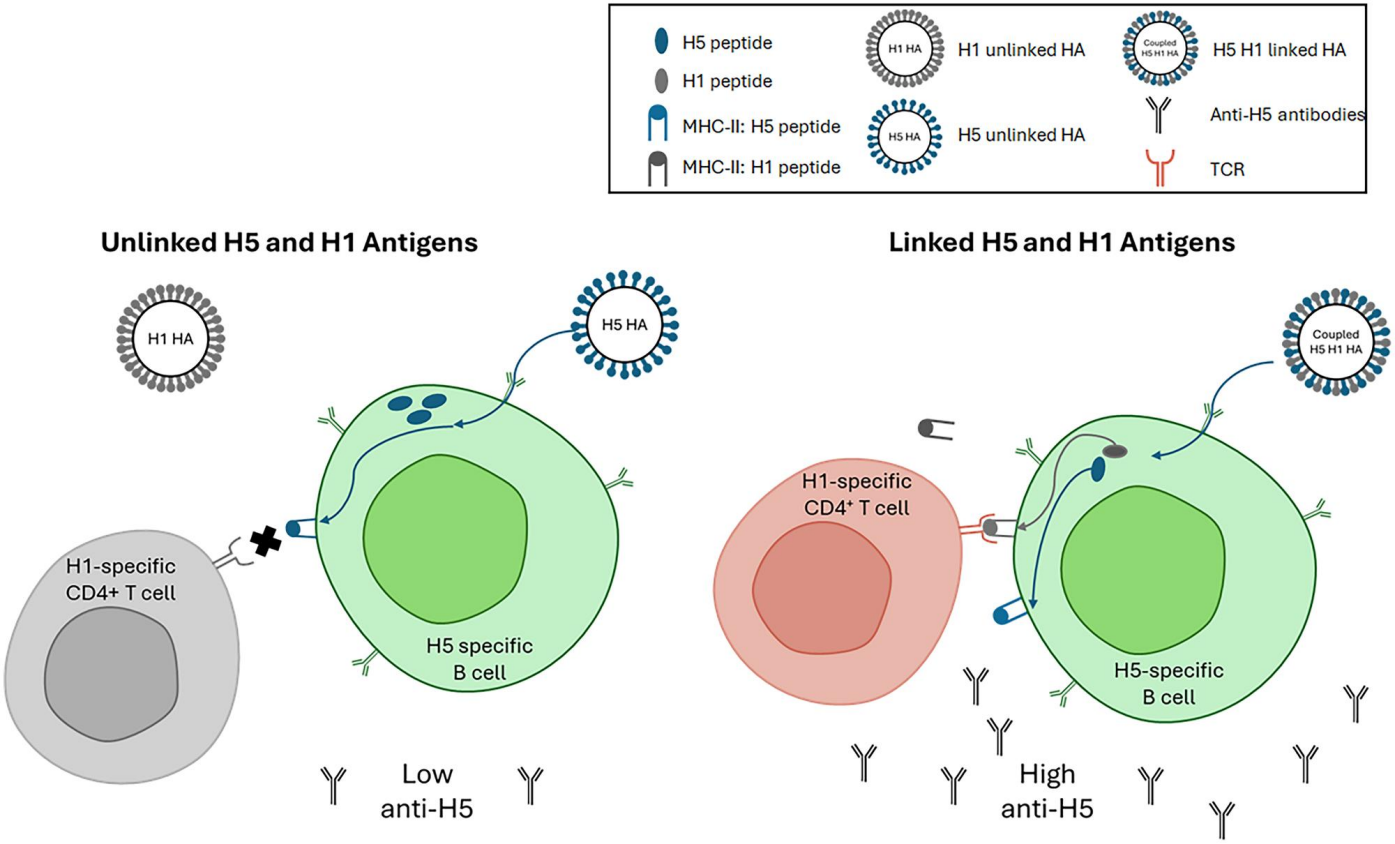
Linked recognition enables heterologous T-cell help to drive enhanced anti-H5 antibody responses. H5-specific B cells bind the H5 portion of an H5–H1 conjugate, internalize the complex, and present both H5- and H1-derived peptides on MHC-II. H1-specific CD4^+^ T cells recognize the H1 peptide–MHC-II complex via the T cell receptor (TCR), leading to productive T–B cell collaboration and robust anti-H5 antibody production. CD40L–CD40 costimulatory signaling, which is required for full B cell activation and class switching, is not shown.

Mallajoysula et al. recently demonstrated that antigen coupling can “borrow” T cell help to boost responses to challenging antigens such as H5N1 HA.^55^ By covalently linking H5N1 HA with a more common seasonal HA (eg H1), B cells specific for H5 can internalize the entire complex, process both HAs, and present H1-derived peptides on MHC-II (Fig. 2). This allows pre-existing H1-specific CD4^+^ T cells to provide help to H5-binding B cells, a form of linked recognition that recruits memory T-cell help from heterologous strains. This strategy markedly enhances anti-H5 responses compared with unlinked antigen cocktails, demonstrating the ability of antigen coupling to overcome MHC-II restrictions and amplify subdominant responses to avian influenza antigens (Fig. 2).

## High-throughput testing in human immune organoids

We recently expanded the immune organoid platform to include human spleen tissue using a protocol developed by Kathuria et al. (submitted). Unlike tonsils, which consist predominantly of B and T cells, the spleen contains a more diverse immune cell population, including monocytes, macrophage, NK cells, and dendritic cells. Leveraging this system, we performed the first high-throughput screen of cytokine adjuvants in vaccine-induced antibody responses. Using a low-cell-input protocol, hundreds of organoids were generated in a 96-well format.^54^ The screen tested 19 recombinant human cytokines at three concentrations each (1, 10, and 100 ng/ml), co-administered with IIV. Responses were measured on day 7 with 5 biological replicates per condition.

The high-throughput screen in human spleen and tonsil organoids systematically evaluated 19 cytokines for their capacity to augment antibody responses to inactivated influenza vaccine (IIV). The study identified multiple potent cytokine adjuvants, including all three Type I interferons (IFN-α, IFN-β, IFN-ω), IL-21, IL-12, IL-9, and IL-10, with effects detected at picomolar concentrations. However, IFN-γ did not enhance IIV-induced antibodies in this functional system. NULISA cytokine profiling of IIV- and live attenuated influenza vaccine (LAIV)-stimulated spleen organoids revealed that LAIV uniquely induced a broad Type I and III interferon response, alongside IL-10, IL-12, and other immunoregulatory cytokines. While both the inactivated and live attenuated vaccines induce a strong Type II IFN response (IFN-γ), the inactivated influenza vaccine failed to trigger Type I IFNs. Adding IFN-β to IIV-treated organoids reproduced most of the LAIV-specific cytokine induction profile, including upregulation of other Type I IFNs and IL-10, and boosted antibody production to LAIV-comparable levels, indicating that Type I IFN signaling is a central driver of the live vaccine immune program.

In parallel, the analysis identified a second, Type I IFN-independent cytokine pathway centered on IL-12 and IL-21. IL-12 induced IL-21 production in spleen organoids consistent with their roles in T follicular helper cell activation and direct B cell modulation, respectively.^56^ Both cytokines were strongly induced by LAIV but not by IFN-β, indicating that they are orthogonal to the Type I IFN cascade. This dual-pathway model, Type I IFN-driven versus IL-12/IL-21, highlights distinct but complementary mechanisms by which cytokines can enhance vaccine-induced humoral immunity.

These results demonstrate that high-throughput screening in immune organoids can evaluate the functionality of many molecules in parallel and identify the most potent hits for further functional testing. Meanwhile, the systemic investigation also allowed a more thorough pathway analysis than a typical low-throughput approach.

## Future: a functional systems immunology approach

When naive B cells are used as the sole source of B cells, tonsil organoids still produce high-affinity antibodies against an influenza antigen, demonstrating de novo affinity maturation.^49^ However, these organoids are limited in their ability to mount naïve responses against the yellow fever vaccine, while our recent findings suggest that spleen organoids can initiate a more robust IgM response to yellow fever (Elsa Sola, unpublished results). These disparities likely reflect the fact that spleens contain far higher frequencies of antigen presentation cells such as monocyte, macrophage and dendritic cells (unpublished results). Further studies should focus on developing strategies to promote strong de novo responses in spleen organoids.

Over the past decade, systems immunology has enabled the high-dimensional characterization of influenza vaccine responses in humans using technologies such as RNA-seq,^57–65^ mass cytometry,^66^ and cytokine profiling.^66^ While powerful, these approaches are primarily correlative and limited in establishing causality. The advent of human immune organoids enables direct hypothesis testing in a human system.^49^ High-throughput screening further expands this capacity, allowing massively parallel experiments. In our initial screen, we measured flu-specific IgG as a single output.^54^ Future studies can incorporate multiple phenotypic readouts using systems immunology technologies. The combination of functional screening and high-dimensional profiling offers a transformative approach (functional systems immunology) for mechanistic insight and immunotherapeutic innovation.

## Conclusion

Human immune organoid platforms represent a transformative advance in vaccine research and immunological discovery. As we face increasing challenges from pandemic threats such as avian influenza, harnessing such innovative technologies will empower vaccine development. Traditionally, only a limited number of vaccine designs can be evaluated in animal models within a single study. By contrast, high-throughput screening using human spleen organoids enables simultaneous testing of a large panel of vaccine candidates, rapidly identifying those with optimal immunogenicity. Subsequent validation in animal models can then focus on confirming protective efficacy, particularly survival benefit, of the top-performing candidates. This integrated approach dramatically expands the search space for rational vaccine design.

## Data Availability

No new data were generated or analyzed for this review.
